# Lipopolysaccharide from *Rhodobacter sphaeroides* (TLR4 antagonist) attenuates hypersensitivity and modulates nociceptive factors

**DOI:** 10.1080/13880209.2018.1457061

**Published:** 2018-04-16

**Authors:** Agnieszka M. Jurga, Ewelina Rojewska, Wioletta Makuch, Joanna Mika

**Affiliations:** Department of Pain Pharmacology, Institute of Pharmacology, Polish Academy of Sciences, Krakow, Poland

**Keywords:** Chronic constriction injury, cytokines, LPS-RSU

## Abstract

**Context:** Accumulating evidence has demonstrated that Toll-like receptors (TLRs), especially TLR4 localized on microglia/macrophages, may play a significant role in nociception.

**Objective:** We examine the role of TLR4 in a neuropathic pain model. Using behavioural/biochemical methods, we examined the influence of TLR4 antagonist on levels of hypersensitivity and nociceptive factors whose contribution to neuropathy development has been confirmed.

**Materials and methods:** Behavioural (von Frey’s/cold plate) tests were performed with Wistar male rats after intrathecal administration of a TLR4 antagonist (LPS-RS ULTRAPURE (LPS-RSU), 20 μG: lipopolysaccharide from *Rhodobacter sphaeroides*, InvivoGen, San Diego, CA) 16 H and 1 h before chronic constriction injury (cci) to the sciatic nerve and then daily for 7 d. three groups were used: an intact group and two cci-exposed groups that received vehicle or LPS-RSU. tissue [spinal cord/dorsal root ganglia (DRG)] for western blot analysis was collected on day 7.

**Results:** The pharmacological blockade of TLR4 diminished mechanical (from ca. 40% to 16% that in the INTACT group) and thermal (from ca. 51% to 32% that in the INTACT group) hypersensitivity despite the enhanced activation of IBA-1-positive cells in DRG. Moreover, LPS-RSU changed the ratio between IL-18/IL-18BP and MMP-9/TIMP-1 in favour of the increase of antinociceptive factors IL-18BP (25%-spinal; 96%-DRG) and TIMP-1 (15%-spinal; 50%-DRG) and additionally led to an increased IL-6 (40%-spinal; 161%-DRG), which is known to have analgesic properties in neuropathy.

**Conclusions:** Our results provide evidence that LPS-RSU influences pain through the expression of TLR4. TLR4 blockade has analgesic properties and restores the balance between nociceptive factors, which indicates its engagement in neuropathy development.

## Introduction

Neuropathic pain is the pathological, prolonged, excessive sensing of stimuli connected with mechanical nerve injury or a co-occurring illness. Unfortunately, there is still no satisfactory treatment for this disorder. The involvement of immune cells, including microglia, in neuropathy and opioid effectiveness is well reported (DeLeo and Yezierski 2001; Beggs and Salter 2013; Chen et al. 2014; Leduc-Pessah et al. 2017). Based on the data from animal models, microglia/macrophages are upregulated almost immediately after nerve injury and contribute to the development of neuropathic pain. Astrocyte activation occurs after microglia/macrophage activation; however, it persists for approximately 12 weeks after the injury (Colburn et al. 1999; Tanga et al. 2004). In the early phase of neuropathic pain, the activated microglia/macrophages release pro- (e.g., iNOS, IL-1β, IL-18, TNFα, and MMP-9) and anti- (e.g., IL-1Ra, IL-10, IL-18BP, and TIMP-1) nociceptive factors. The disrupted balance between these factors is well described (Mika 2008; Rojewska, Popiolek-Barczyk, et al. 2014). The contributions of these specific factors to the mechanism underlying the development of neuropathic pain are still not completely understood, but the role of microglial/macrophage activation in neuropathic pain is undisputed.

According to the results obtained by our group and other laboratories, minocycline, a p38 MAPK/MMP-9 inhibitor, has analgesic properties and diminishes the activation of microglia/macrophages in neuropathic pain models (Tikka et al. 2001; Mika et al. 2007; Cui et al. 2008; Hutchinson et al. 2008). In addition, other inhibitors that affect intracellular pathways, such as SB203580 (p38 MAPK) (Tsuda et al. 2004), LY294002 (PI3K) (Yu et al. 2012), or parthenolide (NFκB) (Popiolek-Barczyk et al. 2015), diminish microglial/macrophage activation, the levels of nociceptive factors, and pain-related behaviours. Based on their direct association with this issue, the roles of many microglial/macrophage receptors in the pathological mechanisms underlying neuropathic pain are being investigated (Bhangoo et al. 2007; Beggs and Salter 2013; Lewis et al. 2013). The expression of numerous surface receptors, e.g., receptors for interleukins (IL-1R and IL-18R) or chemokines (CCR2 and CCR5), exhibits changes in response to neuropathic pain, and our results show that their blockade diminishes neuropathic pain (Pilat et al. 2015, 2016; Kwiatkowski et al. 2016; Piotrowska et al. 2016). Among others, Toll-like receptors (TLRs) are proposed to play important roles in neuropathic pain processes (Christianson et al. 2011; Liu et al. 2012). Subtype 4 (TLR4) has been a particular focus, and its contributions have been investigated, e.g., using TLR4 knockout mice, which do not develop neuropathy (Bettoni et al. 2008). Moreover, paw injections of a TLR4 ligand (LPS, lipopolysaccharide) provoke pain-related behaviour (Calil et al. 2014), and intrathecal (*ith.*) administration of a TLR4 antagonist (LPS-RS Ultrapure, LPS-RSU) attenuates pain and enhances buprenorphine-induced analgesia, as shown in our previous report (Jurga, Rojewska, et al. 2016). Importantly, TLR4 is expressed on microglia/macrophages (Lehnardt et al. 2003).

It has already been shown that direct TLR4 activation modulates some factors involved in nociception, such as IL-1β (Calil et al. 2014). We have decided to investigate the putative changes in the levels of the pro- and antinociceptive factors released by activated microglia/macrophages that are typically disrupted in neuropathic pain models (Rojewska, Popiolek-Barczyk, et al. 2014). Using Western blotting, we estimated the influence of repeated intrathecal administration of LPS-RSU on microglial/macrophage and astroglial activation and the levels of nociceptive factors (IL-1β, IL-1Ra, IL-18, IL-18BP, IL-6, IL-10, MMP-9, and TIMP-1) in the spinal cord and DRG during the development of neuropathic pain.

## Materials and methods

### Animals

Wistar male rats weighing between 290 and 330 g (Charles River Laboratories, Sulzfeld, Germany) were kept in acrylic cages with sawdust (12 h light/dark cycle at room temperature), with free access to water and rat chow pellets. Only the minimal essential number of animals was used in experiments, which were performed according to the recommendations of IASP (Zimmermann 1983), the NIH Guide for the Care and Use of Laboratory Animals, and in accordance with the recommendations of the 2nd Local Ethical Committee on Animal Testing in the Institute of Pharmacology, PAS (Krakow, Poland); permission number: 1277.

### Intrathecal implantation

Intrathecal (*ith*.) administration of substances was achieved by implanting catheters according to the method described earlier (Yaksh and Rudy 1976). The procedure was performed under pentobarbital (60 mg/kg; *i.p.*) anaesthesia. The polyethylene catheters (PE 10, Intramedic; Clay Adams, Parsippany, NJ) were sterilized before the operation in 70% (v/v) EtOH and flushed with water for the injection directly before insertion. Rats were placed on a stereotaxic table (David Kopf Instruments, Tujunga, CA), and an incision in the atlantooccipital membrane was made for the introduction of the catheter (7.8 cm long) into the subarachnoid space. After insertion, the catheters were slowly flushed with 10 µL of water for the injection, and the tip was tightened for separation from the environment. After implantation, the rats were observed for physical impairments and allowed to recover for a minimum of 1 week before the actual experiment.

### Neuropathic pain model

Right sciatic nerve ligation was performed in rats (Bennett and Xie 1988) under pentobarbital anaesthesia (60 mg/kg; *i.p.*) as described in our previous study (Kwiatkowski et al. 2016; Pilat et al. 2016; Jurga et al. 2017). The biceps femoris and gluteus superficialis were separated, and the right sciatic nerve was exposed. Four ligatures (4/0 silk) were placed around the nerve with 1 mm spacing until they elicited a brief twitch in the right hind limb. In every case, the surgery caused neuropathic pain behaviour on day 2, such as mechanical and thermal hypersensitivity.

### Pharmacological treatment and experimental groups

Animals were divided into three experimental groups: *INTACT*: healthy, non-operated rats; *V*: vehicle-treated rats after chronic constriction injury (CCI); and *LPS-RSU*: LPS-RS Ultrapure-treated rats after CCI. LPS-RSU (20 µg/5 µL; dissolved in water for injection), a TLR4-specific antagonist derived from *Rhodobacter sphaeroides* (InvivoGen, San Diego, CA), was administered at the dose chosen in our previous study (Kwiatkowski et al. 2016; Pilat et al. 2016; Jurga et al. 2017). The antagonist or vehicle was administered by *ith*. injection once per day. The first injection was administered 16 h before the CCI and the second 1 h before the CCI. Then, seven consecutive injections were administered for the next 7 d, starting the day after the CCI (CCI surgery was defined as day 0; substances were administered from day 1 until day 7). Animals received nine injections during the experiment (two pre-emptive injections before the CCI and then seven injections after the CCI). Injections were performed using a 50 µL Hamilton syringe; 5 µL of substance was injected per animal, followed by injection of 10 µL of water. This method of administration is referred to as ‘repeated administration’ throughout the text.

### Hypersensitivity assessment tests

Two behavioural tests, von Frey’s and the cold plate test, were performed on days 2 and 7 after the CCI, 2 h after the morning LPS-RSU administration. The control groups received vehicle (water for injections) according to the same schedule.

### Mechanical hypersensitivity

The mechanical nociceptive threshold was determined using an automatic von Frey apparatus (Dynamic Plantar Aesthesiometer, Ugo Basile, Italy) as previously described by our laboratory (Makuch et al. 2013). Rats were placed in plastic cages with a wire net floor and von Frey’s filament was applied to the midplantar surface of the on the right hind paw. The mass (in grams) required to induce a response represented the nociceptive threshold. Measurements were taken automatically with a maximum stimulus of 26 g.

### Thermal hypersensitivity

Thermal hypersensitivity was assessed using a Cold/Hot Plate Analgesia Meter (Columbus Instruments, Columbus, OH), as described in our previous study (Rojewska, Popiolek-Barczyk, et al. 2014). The rats were placed on the cold plate, and the time (in seconds) until the hind paw was lifted was recorded. The temperature (5 °C) of the plate was constant and the maximum time of a single measurement was 30 s. The right foot reaction was always the first.

### Biochemical evaluation of protein levels

Rat ipsilateral dorsal lumbar (L4–L6) spinal cord and dorsal root ganglia (DRG; L4–L6) were collected immediately after decapitation on day 7 post CCI, 6 h after the last substance administration. The three DRGs L4, L5, and L6 from one animal were pooled and treated as one sample. Tissue was stored at −80 °C until processing as described previously (Rojewska, Popiolek-Barczyk, et al. 2014; Jurga, Piotrowska, et al. 2016). Tissues were homogenized in RIPA lysis buffer containing protease inhibitors and cleared by centrifugation (14,000 rpm for 30 min, 4 °C). After denaturation in loading buffer (4× Laemmli Sample Buffer, Bio-Rad, Hercules, CA) for 8 min at 98 °C, 20 μg of protein was loaded and resolved on Criterion™ TGX™ precast polyacrylamide gradient gels (Bio-Rad, Hercules, CA). Proteins were transferred to Immune-Blot PVDF membranes (Bio-Rad, Hercules, CA) using semi-dry transfer (30 min, 25 V), blocked for 1 h at room temperature using 5% non-fat dry milk (Bio-Rad, Hercules, CA) in Tris-buffered saline with 0.1% Tween 20 (TBST), washed in TBST, and then incubated overnight at 4 °C with primary antibodies targeting the following proteins: TLR4 (Santa Cruz Biotechnology, Santa Cruz, CA #sc-293072, 1:500), IBA-1 (Proteintech #10904-1-AP, 1:1000), GFAP (Santa Cruz Biotechnology, Santa Cruz, CA #sc-6171, 1:1000), IL-1β (Abcam, Cambridge, UK #ab9787, 1:1000), IL-1Ra (Abcam, Cambridge, UK #ab175392, 1:1000), IL-18 (Abcam, Cambridge, UK #ab191860, 1:1000), IL-18BP (Novus Biologicals, Cambridge, UK #NB110-57117, 1:1000), IL-6 (Invitrogen, Carlsbad, CA #ARC0062, 1:500), IL-10 (Invitrogen, Carlsbad, CA #ARC17536, 1:1000), MMP-9 (EMD Millipore, Billerica, MA #AB19016, 1:1000), TIMP-1 (Novus Biologicals, Cambridge, UK #NBP1-96554, 1:1000), and GAPDH (EMD Millipore, Billerica, MA #MAB374, 1:5000; as a loading control). Blots were incubated with the corresponding HRP-conjugated secondary polyclonal antibody (Vector Laboratories, Burlingame, CA, peroxidase anti-mouse IgG (H + L) #PI-2000; peroxidase anti-mouse IgG (H + L) #PI-1000, 1:5000) for 1 h at RT. Antibodies were diluted with reagent from a SignalBoost Immunoreaction Enhancer Kit (Merck Millipore, Billerica, MA). Immunocomplexes were detected using Clarity™ Western ECL Substrate (Bio-Rad, Hercules, CA) and visualized using a Fujifilm LAS-4000 fluoroimager system. The blots were stripped using Restore Western Blot Stripping Buffer (Thermo-Scientific, Waltham, MA) for 15 min at RT and reprobed if needed. Representative bands for each group are shown below each column on the respective graph and were obtained from photos of the same membrane.

### Statistical analysis

All measurement data are presented as the means ± SEM, and the statistical analyses were performed using GraphPad Prism 7 software (La Jolla, CA). Behavioural (von Frey/cold plate tests) and protein analyses (Western blotting) were performed on three groups: the INTACT, V-, and LPS-RSU-treated CCI-exposed groups described in detail in the ‘Pharmacological treatment and experimental groups’ section above. One-way ANOVA followed by Bonferroni’s *post hoc* test was used to assess inter-group differences. Significance was defined as follows: *p < 0.05, **p < 0.01, and ***p < 0.001 – differences compared with the INTACT group; #*p <* 0.05, ##*p <* 0.01, and ###*p <* 0.001 – differences compared with the V-treated CCI-exposed group.

## Results

### The influence of repeated *ith.* administration of LPS-RSU on pain-related behaviours after CCI

**Figure 1.**
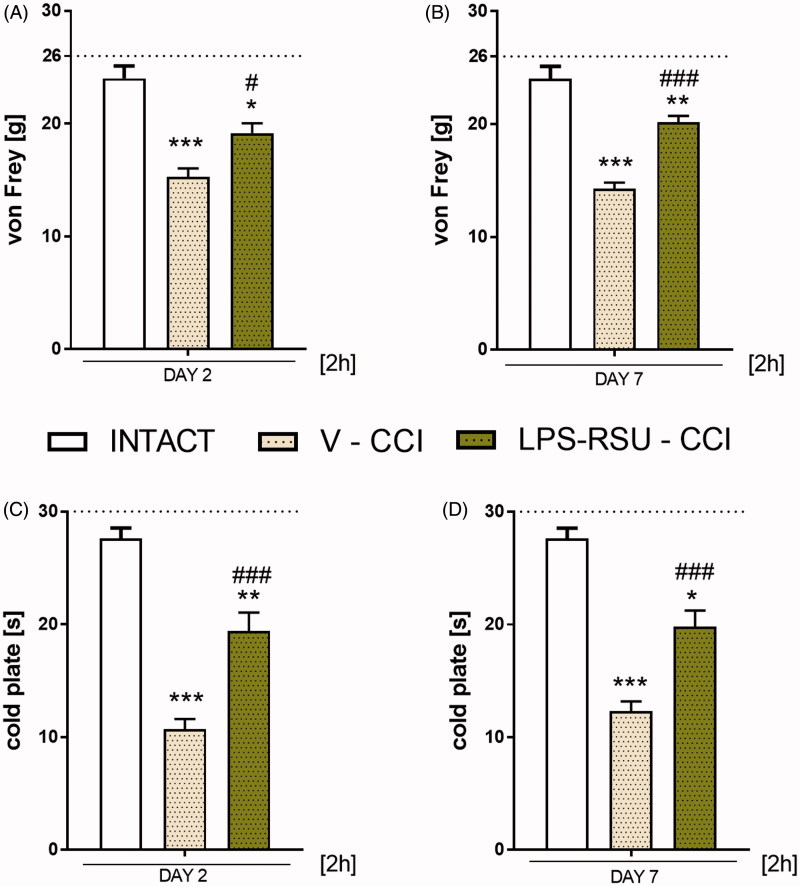
Effects of repeated *ith.* LPS-RSU administration (20 µg/5 µL, *ith.*) on the development of mechanical (A, B; von Frey’s test) and thermal (C, D; cold plate test) hypersensitivity on days 2 (INTACT *n =* 5*;* V-CCI *n =* 18*;* LPS-RSU-CCI *n =* 17) and 7 (INTACT *n =* 5; V-CCI *n =* 22*;* LPS-RSU-CCI *n =* 24) after CCI, as measured 2 h after the last drug administration. The behavioural results are presented as the means ± SEM, and the horizontal dotted line shows the cutoff value (A: 26 g, B: 30 s). The inter-group differences were analyzed using one-way ANOVA with Bonferroni’s multiple comparisons test. ∗*p <* 0.05, ∗∗*p <* 0.01, and ***p < 0.001 indicates a significant difference compared with the INTACT animals; #*p <* 0.05 and ###*p <* 0.001 indicate significant differences compared with the vehicle (V)-treated CCI-exposed group.

Chronic constriction injury to the right sciatic nerve caused pain-related behaviours, as measured on days 2 (Figure 1(A,C)) and 7 (Figure 1(B,D)) after the operation. Repeated administration of LPS-RSU significantly attenuated the mechanical (day 2: *p <* 0.05 Figure 1(A); day 7: *p <* 0.001; Figure 1(B)) and thermal (day 2: *p <* 0.001 Figure 1(C); day 7: *p <* 0.001; Figure 1(D)) hypersensitivity measured 2 h after the morning injection; however, it does not completely reverse hypersensitivity Figure 1(A–D)).

### The influence of repeated *ith*. LPS-RSU administration on TLR4, IBA-1, and GFAP protein levels in the spinal cord and DRG on day 7 after CCI

**Figure 2.**
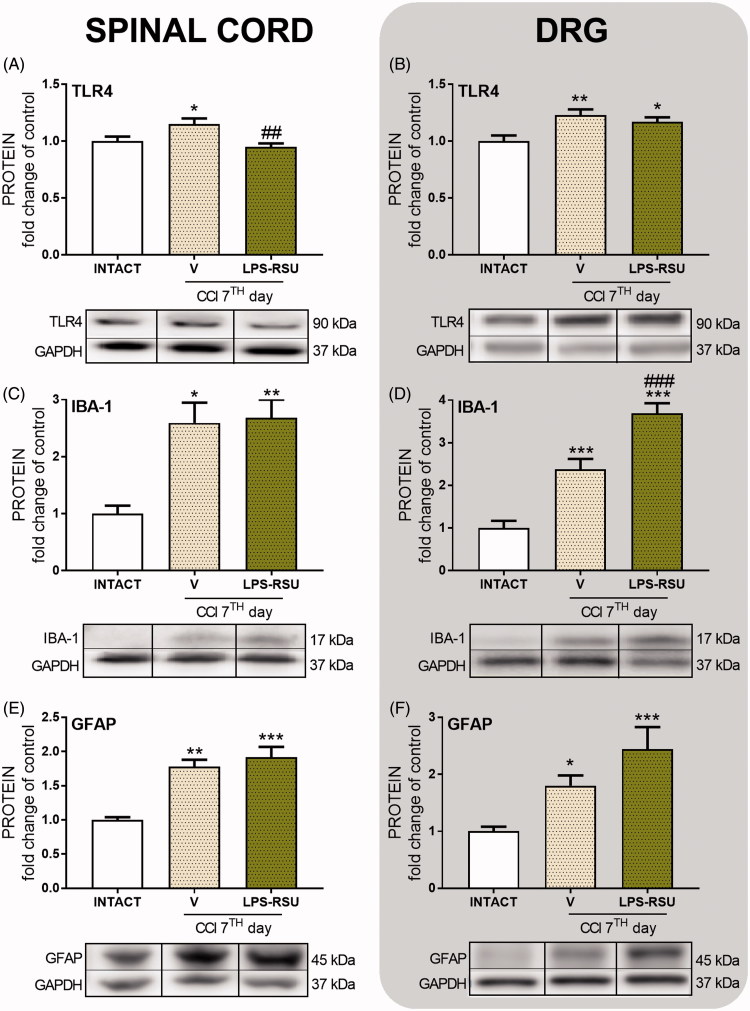
Western blot analysis of the levels of TLR4 (A, *n =* 6/group; B, *n =* 6–8/group), IBA-1 (C, *n =* 4–7/group; D, *n =* 7–8/group), and GFAP (E, *n =* 6–8/group; F, *n =* 10–11/group) proteins in the rat ipsilateral dorsal lumbar spinal cord (A, C, E) and DRG (B, D, F) after repeated *ith.* administration of LPS-RSU (20 µg/5 µL, *ith.*) on day 7 after chronic constriction injury (CCI). The data are presented as the means ± SEM. Inter-group differences were analyzed using one-way ANOVA followed by Bonferroni’s multiple comparisons test. ∗*p <* 0.05, ∗∗*p <* 0.01, and ∗∗∗*p <* 0.001 compared with the INTACT group; ##*p <* 0.01 and ###*p <* 0.001 compared with the vehicle (V)-treated CCI group.

The TLR4 levels in the spinal cord and DRG were significantly increased after the CCI compared with the levels in the INTACT group (*p <* 0.05, Figure 2(A); *p <* 0.01, Figure 2(B)) and were downregulated after repeated administration of LPS-RSU in the spinal cord (*p <* 0.01; Figure 2(A)). The IBA-1 protein levels were significantly increased in both the spinal cord (*p <* 0.05, Figure 2(C)) and DRG (*p <* 0.001, Figure 2(D)). Repeated administration of LPS-RSU caused an increase in IBA-1 level in DRG (*p <* 0.001, Figure 2(D)). GFAP levels were increased in the spinal cord (*p <* 0.01, Figure 2(E)) and DRG (*p <* 0.05, Figure 2(F)) after the CCI compared with those in the INTACT group, but the LPS-RSU treatment did not influence the increased GFAP levels in either structure.

### The influence of repeated *ith*. LPS-RSU administration on IL-1β or IL-1Ra protein levels in the spinal cord and DRG 7 d after CCI

**Figure 3.**
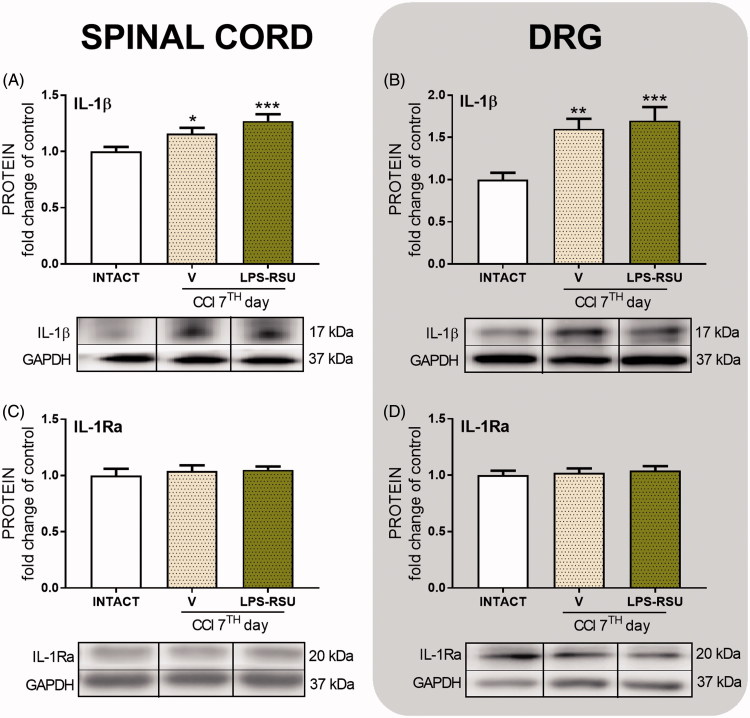
Western blot analysis of the levels of IL-1β (A, *n =* 11–13/group; B, *n =* 10–16/group) and IL-1Ra (C, *n* = 11–14/group; D, *n =* 5–8/group) proteins in the rat ipsilateral dorsal lumbar spinal cord (A, C) and DRG (B, D) after repeated *ith.* administration of LPS-RSU (20 µg/5 µL, *ith.*) on day 7 after chronic constriction injury (CCI). The data are presented as the means ± SEM. Inter-group differences were analyzed using one-way ANOVA followed by Bonferroni’s multiple comparisons test. ∗*p <* 0.05, ∗∗*p <* 0.01, and ∗∗∗*p <* 0.01 compared with the INTACT group.

The protein level of the pronociceptive factor IL-1β was significantly increased in the spinal cord (*p <* 0.05, Figure 3(A)) and DRG (*p <* 0.01, Figure 3(B)) after CCI compared with that in the INTACT group. Repeated administration of LPS-RSU did not influence those levels. The antinociceptive factor IL-1Ra was maintained at the same levels in both structures after the CCI compared with its level in the INTACT group, and LPS-RSU administration had no effect on its levels (Figure 3(C,D)).

### The influence of repeated *ith.* LPS-RSU administration on IL-18 and IL-18BP protein levels in the spinal cord and DRG 7 d after CCI

**Figure 4.**
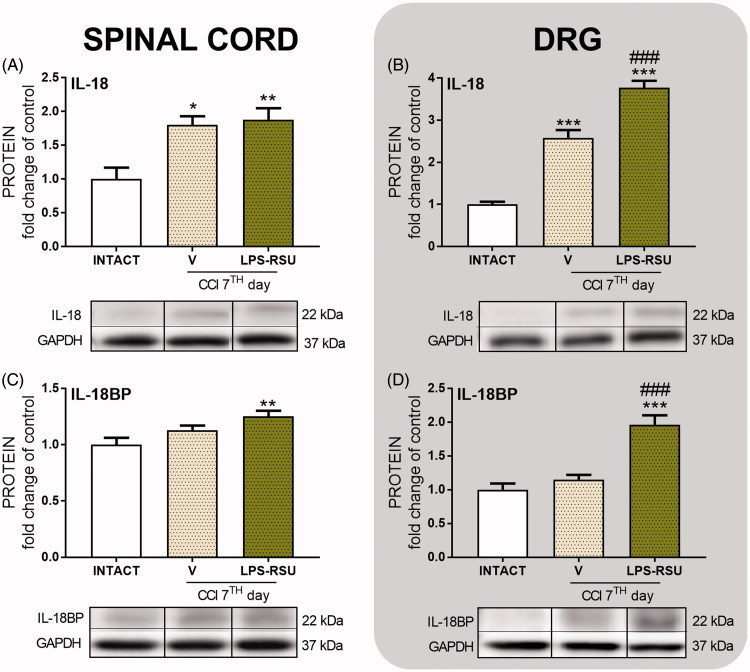
Western blot analysis of the levels of IL-18 (A, *n* = 5–8/group; B, *n =* 4–5/group) and IL-18BP (C, *n =* 12–16/group; D, *n* = 5–7/group) proteins in the rat ipsilateral dorsal lumbar spinal cord (A, C) and DRG (B, D) after repeated *ith.* administration of LPS-RSU (20 µg/5 µL, *ith.*) on day 7 after chronic constriction injury (CCI). The data are presented as the means ± SEM. Inter-group differences were analyzed using one-way ANOVA followed by Bonferroni’s multiple comparisons test. ∗*p <* 0.05, ∗∗*p <* 0.01, and ∗∗∗*p <* 0.001 compared with the INTACT group; ###*p <* 0.001 compared with the vehicle (V)-treated CCI group.

The levels of the pronociceptive protein IL-18 were increased in both the spinal cord (*p <* 0.05, Figure 4(A)) and DRG (*p <* 0.001, Figure 4(B)) after CCI compared with the levels in the INTACT group and were significantly upregulated in DRG after administration of the TLR4 antagonist (*p <* 0.001, Figure 4(B)) compared with those in the V-treated CCI-exposed rats. We did not observe significant changes in the levels of the antinociceptive protein IL-18BP after the CCI (Figure 4(C,D)). The LPS-RSU treatment significantly increased the IL-18BP protein level in the spinal cord (*p <* 0.01, Figure 4(C)) compared with that in the INTACT group and in DRG (*p <* 0.001, Figure 4(D)) compared with that in the V-treated CCI-exposed and INTACT groups.

### The influence of repeated *ith*. LPS-RSU administration on IL-6 and IL-10 protein levels in the spinal cord and DRG 7 d after CCI

**Figure 5.**
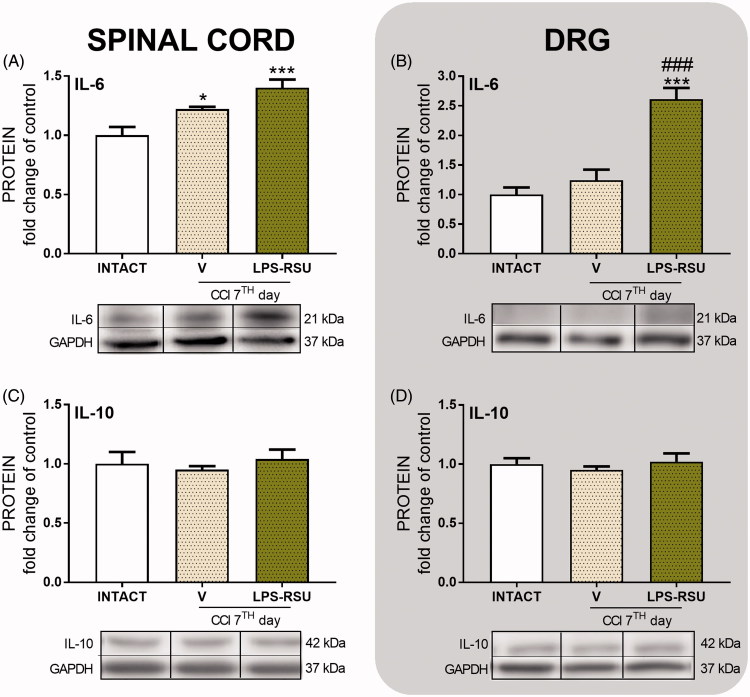
Western blot analysis of the levels of the IL-6 (A, *n* = 5–6/group; B, *n =* 5–8/group) and IL-10 (C, *n =* 4–8/group; D, *n* = 8/group) proteins in the rat ipsilateral dorsal lumbar spinal cord (A, C) and DRG (B, D) after repeated *ith.* administration of LPS-RSU (20 µg/5 µL, *ith.*) on day 7 after chronic constriction injury (CCI). The data are presented as the means ± SEM. Inter-group differences were analyzed using one-way ANOVA followed by Bonferroni’s multiple comparisons test. ∗*p <* 0.05 and ∗∗∗*p <* 0.001 compared with the INTACT group; ###*p <* 0.001 compared with the vehicle (V)-treated CCI group.

The IL-6 protein level was significantly increased in the spinal cord after CCI compared with that in the INTACT group (*p <* 0.05, Figure 5(A)). The LPS-RSU treatment increased the levels of this protein in DRG (*p <* 0.001, Figure 5(B)) compared with those in the INTACT and V-treated CCI-exposed groups. The levels of the IL-10 protein remained unchanged after CCI and LPS-RSU treatment.

### The influence of repeated *ith.* LPS-RSU administration on MMP-9 and TIMP-1 protein levels in the spinal cord and DRG 7 d after CCI

**Figure 6.**
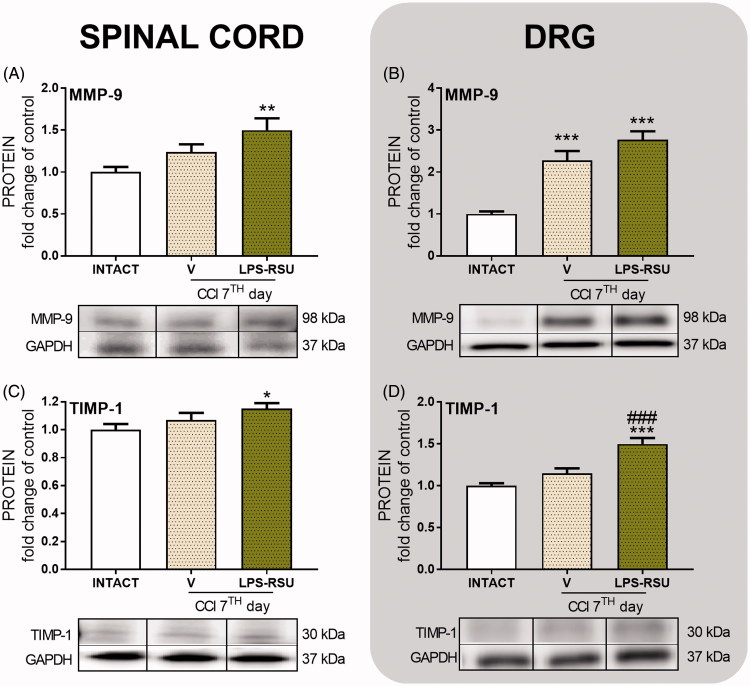
Western blot analysis of the levels of the MMP-9 (A, *n =* 12–14/group; B, *n =* 12–13/group), and TIMP-1 (C, *n =* 11–14/group; D, *n =* 10–12/group) proteins in the rat ipsilateral dorsal lumbar spinal cord (A, C) and DRG (B, D) after repeated *ith.* administration of LPS-RSU (20 µg/5 µL, *ith.*) on day 7 after chronic constriction injury (CCI). The data are presented as the means ± SEM. Inter-group differences were analyzed using one-way ANOVA followed by Bonferroni’s multiple comparisons test. ∗*p <* 0.05, ∗∗*p <* 0.01, and ∗∗∗*p <* 0.001 compared with the INTACT group; ###*p <* 0.001 compared with the vehicle (V)-treated CCI group.

The MMP-9 level was significantly upregulated in DRG (*p <* 0.001, Figure 6(B)) in the V-treated CCI-exposed group compared with that in the INTACT group, but the level was not affected by repeated administration of LPS-RSU. The TIMP-1 protein levels were not changed in the V-treated CCI-exposed group (Figure 6(C,D)) compared with those in the INTACT group, but the LPS-RSU treatment increased TIMP-1 levels in DRG (*p <* 0.001, Figure 6(D)) compared with those in the V-treated CCI-exposed group.

## Discussion

**Scheme 1.**
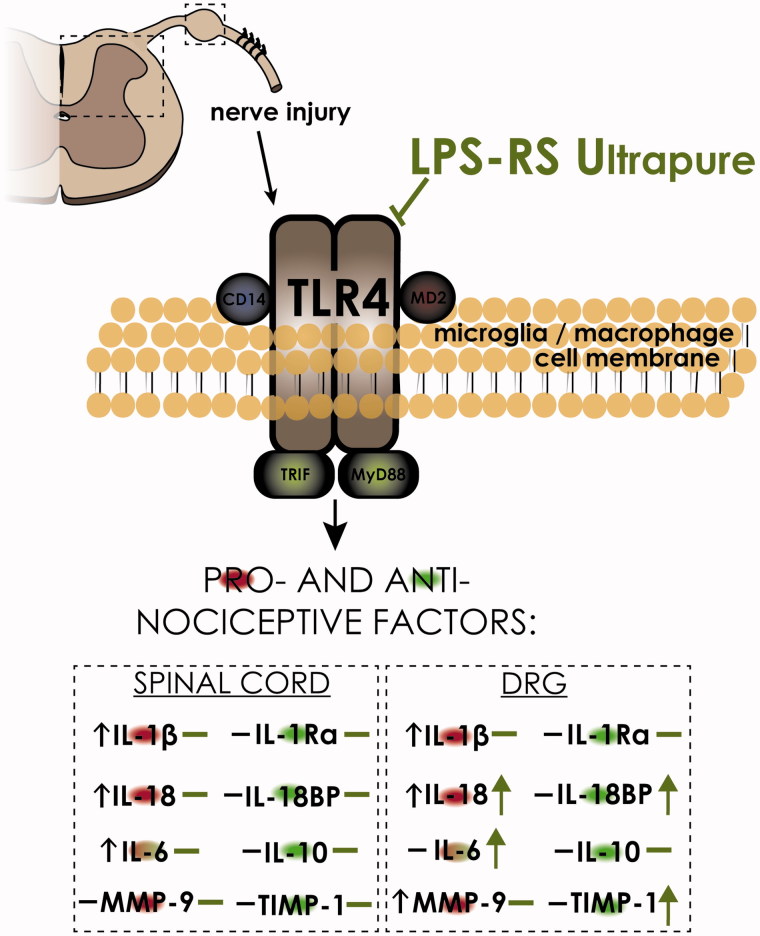
LPS-RSU influences IBA-1-positive cells and certain nociceptive factors on day 7 after CCI. Our data strongly support the hypothesis that TLR4 plays a significant role in neuropathy. In the nervous system, TLR4 is expressed on microglia/macrophages (Bandow et al. 2012), and pre-emptive repeated administration of the TLR4 antagonist LPS-RSU potentiates the increase in the number of IBA-1-positive cells in the DRG after chronic constriction injury (CCI). Moreover, LPS-RSU induces a change in the ratio between IL-18/IL18BP and MMP-9/TIMP-1, in favour of the antinociceptive neuropathic factors IL-18BP and TIMP-1. Additionally, LPS-RSU administration increased the IL-6 level, which under some circumstances is known to have analgesic properties. In summary, pharmacological blockade of TLR4 diminished hypersensitivity and modulated the levels of nociceptive proteins.

Based on the data presented in this study, the attenuation of pain symptoms observed after LPS-RSU administration is related to the modulation of IBA-1-positive cell activity in DRG and not in the spinal cord as we assumed at the beginning of the experiments. Moreover, a highly specific TLR4 antagonist modulated the interleukin expression levels in DRG, restoring the balance between pronociceptive IL-18 and analgesic IL-18BP, and increased the IL-6 level, which in neuropathy is known to have analgesic properties (Gruol and Nelson 1997). Additionally, LPS-RSU induced a change in the ratio between MMP-9 and TIMP-1 in favour of the antinociceptive neuropathic protein TIMP-1. Most of the changes were observed in DRG, and thus, we hypothesize that LPS-RSU influences IBA-1-positive cells, mainly macrophages (Scheme 1).

Recently, accumulating evidence has shown using Western blot and/or immunohistochemical analysis that glial cell activation and neuroinflammation are critical for the development and maintenance of persistent pain (DeLeo and Yezierski 2001; Austin and Moalem-Taylor 2010; Mika et al. 2013). In our previously published studies, we demonstrated an increase in the activation of microglia/macrophages on day 7 after sciatic nerve injury in the lumbar spinal cord and/or in the DRG (Mika et al. 2009, 2010; Rojewska, Korostynski, et al. 2014). Other studies revealed microglia activation on day 2 following sciatic nerve injury, with its highest activation being observed between days 7 and 10 (Kreutzberg 1996; Marchand et al. 2005; Austin and Moalem-Taylor 2010; Bartel and Finger 2013); therefore, we choose day 7 for our Western blot analysis. Using microarray analysis of gene expression for T-cell (Cd3g, Cd3e, Cd3d, CD4, and CD8), B-cells (CD19), and NK-cells (CD335) markers, it was shown that there is no activation or infiltration of those cells into the spinal cord (Rojewska, Korostynski, et al. 2014). It is known that peripheral nerve injury leads to unilateral and strong microglial/macrophage activation in the spinal cord and DRG (Lehnardt et al. 2003; Bishay et al. 2010; Kang et al. 2015) is directly connected with the enhanced expression of various nociceptive factors and receptors (Inoue 2006; Rojewska et al. 2016). These changes disrupt the balance between pronociceptive factors, whose levels become elevated, and antinociceptive factors, which remain unchanged (Rojewska, Popiolek-Barczyk, et al. 2014). According to the literature, including our own research, there is a strong reason to believe that microglia/macrophages are involved in neuropathic pain development in animal models (Yao et al. 1992; Hains and Waxman 2006; Bartel and Finger 2013). Our results confirm that strong IBA-1/GFAP-positive cell activation occurs in the rat CCI model on day 7 after the operation, which has also been observed in many other neuropathic pain models, such as sciatic nerve ligation (Jiang et al. 2016), partial sciatic nerve ligation (Xu et al. 2007), and spared nerve injury (Vega-Avelaira et al. 2007). After peripheral nerve injury, at the spinal cord and/or DRG level, the microglia/macrophages are the first cell type to be activated (Mika et al. 2009). It has been shown that pentoxifylline (Mika et al. 2007), propentofylline (Gallo et al. 2015) as well as blockade of the microglial receptors P2X4R (Zhou et al. 2014; Jurga, Piotrowska, et al. 2016 Jurga et al. 2017), CCR5 (Kwiatkowski et al. 2016), or CCR2 (Piotrowska et al. 2016) can reduce IBA-1-positive cell activation and had analgesic effects parallel to those observed with the aforementioned drugs. However, it is not always the case that activation of microglia is the most beneficial pharmacological strategy. In 2015, it was shown that parthenolide (Popiolek-Barczyk et al. 2015) attenuates neuropathic pain behaviour but enhances IBA-1 cell activation, which is advantageous because it strongly promotes M2 microglia/macrophage polarization. The new strategy for neuropathic pain therapy is to stimulate endogenous antinociceptive factors, which is more physiological than completely abrogating pronociceptive machinery. The LPS-RSU treatment did not influence spinal IBA-1 cell activation but enhanced activation in the DRG. Interestingly, the majority of changes were also observed in the DRG, such as upregulation of the antinociceptive factors IL-18BP, IL-6, and TIMP-1. IBA-1 is a protein marker for both microglia (spinal cord) and macrophages (spinal cord/DRG). With this knowledge, we can assume that the changes in IBA-1 protein level observed in DRG but not in the spinal cord indicate that macrophages and not microglia are responsible for the modulated expression of the measured nociceptive factors.

Microglia/macrophages express TLR4 (Lehnardt et al. 2003; Holm et al. 2012). The LPS-RSU treatment prevented the TLR4 upregulation in the spinal cord that we observed after CCI and that has been previously reported in neuropathy (Ghasemzadeh et al. 2016). Interestingly, our results show TLR4 changes that are not parallel to IBA-1 changes; however, IBA-1 is a marker of microglial/macrophage activation, and thus, cell activity can change independently of the TLR4 level on the surface. Furthermore, in our primary microglial cell cultures, we observed an increase in IBA-1 protein level after LPS stimulation with a significant *downregulation* of TLR4 protein (Popiolek-Barczyk et al. 2017). Therefore, despite the undoubted microglia-TLR4 relationship, the tendency for changes in these two factors (IBA-1 and TLR4) is not necessarily parallel.

Administration of LPS-RSU attenuates mechanical and thermal hypersensitivity (Jurga, Rojewska, et al. 2016), and similar data regarding the pharmacological deactivation of TLR4 signalling have been obtained in many models of pain, such as the paclitaxel-related chemotherapy-induced peripheral neuropathy (CIPN) model, in which LPS-RS also produced analgesia in Sprague–Dawley rats (Li, Zhang, Zhang, et al. 2014), or the cancer-induced bone pain (CIBP) model in Wistar rats (Li et al. 2013). In a spinal cord compression injury model, Sprague–Dawley rats were treated with intraperitoneal TAK-242 (TLR4 antagonist), which reduced pain at low doses (Zhang et al. 2015; Zhao et al. 2015). Ligustilide was used in a complete Freund’s adjuvant (CFA) model and reduced pain in a TLR4-dependent manner (Qian et al. 2016). These reports undoubtedly confirm that TLR4 activation contributes to pain symptoms in various animal models. Moreover, injections of LPS, an agonist of TLR4, into the mouse paw produce pain symptoms (Calil et al. 2014). In addition, intra-articular LPS injections produce weaker hyperalgesia in TLR4 knockout mice than in wild-type C57BL/6 mice (Guerrero et al. 2016), confirming the presumed requirement for the activation of this receptor in pain development.

The other consequence of LPS action following intraplantar injections in mice is increased IL-1β levels in subcutaneous paw tissue (Calil et al. 2014). In the CNS, the main sources of IL-1β biosynthesis are microglia and macrophages, which are highly activated in response to a nerve injury (Yao et al. 1992; Perry and Teeling 2013; Xu et al. 2014; Liu et al. 2017). Intrathecal administration of IL-1β induces hypersensitivity in rats (Malcangio et al. 1996; Oprée and Kress 2000; Mika et al. 2008), which confirms its clear role in neuropathic pain. In the present study, the level of IL-1β protein was also significantly increased after CCI; however, LPS-RSU did not prevent that increase in our model. In contrast, intrathecal administration of IL-1Ra attenuated the development of neuropathic pain (Sweitzer et al. 2001; Mika et al. 2008; Pilat et al. 2015). A lack of IL-1Ra regulation induced by CCI or SNI in rats and mice has been reported (del Rey et al. 2011; Pilat et al. 2015), and is in agreement with our studies. Surprisingly, the IL-1Ra levels in the spinal cord and DRG were not altered by the LPS-RSU treatment. We also studied the level of IL-10, which according to recent studies is one of the most powerful endogenous regulators of pronociceptive cytokine function during the development of neuropathic pain (Moore et al. 1993; Bethea et al. 1999; Brewer et al. 1999; Sawada et al. 1999; Plunkett et al. 2001; Yu et al. 2003; Abraham et al. 2004; Milligan et al. 2006, 2012; Ledeboer et al. 2007; Rojewska et al. 2015). However, our studies present evidence that repeated administration of LPS-RSU did not influence the IL-10 levels in the spinal cord or in DRG.

Further studies have shown that LPS-RSU regulates other important nociception factors in DRG. Since 2008, it has been known that IL-18/IL-18R play a significant role in neuropathy development (Miyoshi et al. 2008; Li, Zhang, Ji, et al. 2014). Intrathecal administration of IL-18 leads to the development of hyperalgesia (Verri et al. 2007). The increase in microglial IL-18 expression is further enhanced by LPS administration, and thus, the level of IL-18 released by activated cells depends on the activity of TLR4 (Miyoshi et al. 2008). Anti-IL-18 antibodies partially attenuate hyperalgesia caused by a nerve injury. Our results revealed a significant increase in the IL-18 level in both structures after CCI, and interestingly, further upregulation after administration of the TLR4 antagonist in DRG. IL-18 is regulated by its endogenous inhibitor, IL-18BP (Kim et al. 2000). IL-18BP binds to IL-18 at a 1:1 ratio and blocks its activity by preventing its binding to the receptor, making it a natural, endogenous inhibitor of this important pronociceptive factor (Kim et al. 2000; Dinarello and Fantuzzi 2003). The neutralization of IL-18 with IL-18BP powerfully reduces inflammation (Plater-Zyberk et al. 2001; Carrascal et al. 2003). As shown in our previous study (Pilat et al. 2016), IL-18BP injections strongly diminish pain in rats subjected to CCI. In the present paper, CCI increased IL-18 levels on day 7 after the injury, whereas the IL-18BP levels remained unchanged, which is in agreement with previously published papers (Pilat et al. 2016). Importantly, repeated *ith.* administration of LPS-RSU led to a significant upregulation of both the pronociceptive protein IL-18 and the antinociceptive protein IL-18BP in the DRG. This upregulation seemed to restore the balance between these factors, and their binding silenced the effects of both proteins, despite their elevated levels, which may be one of the reasons why LPS-RSU has analgesic properties.

Many studies, including the ones conducted by our group, report increased IL-6 levels in the spinal cord and DRG following a peripheral nerve injury (Mika et al. 2008; Brázda et al. 2013; Dubový et al. 2013; Xu et al. 2014). IL-6 is an important mediator of the neuroimmune response (Kreutzberg 1996). Most neuropathy studies indicate that IL-6 is a pronociceptive factor; however, IL-6 has well-documented neuroprotective activity and participates in neuronal differentiation, growth and survival (Gruol and Nelson 1997). IL-6 induces BDNF expression (Murphy et al. 2000), promotes regeneration of injured hypoglossal nerves in mice, and promotes a quicker recovery after traumatic brain injury (Swartz et al. 2001; Penkowa et al. 2003). Intrathecal administration of IL-6 in INTACT animals causes hypersensitivity (DeLeo et al. 1996), but in a 2003 study, Flatters et al. showed that IL-6 in neuropathy has an analgesic effect. As presented in our studies, the CCI-induced upregulation of IL-6 is enhanced in DRG by repeated administration of LPS-RSU, and thus, it may have some analgesic properties as was suggested (Flatters et al. 2003). However, this phenomenon needs to be elucidated.

Among the examined nociceptive mediators, both MMP-9 and TIMP-1 upregulation became significant after repeated LPS-RSU administration in the spinal cord compared with the INTACT group. Our results are in agreement with other results showing that MMP-9 is upregulated after CCI in rats and partial sciatic nerve ligation (PSNL) in mice, and its inhibitor was shown to have analgesic properties in a rat model of spinal nerve ligation (SNL) (Rojewska, Popiolek-Barczyk, et al. 2014; Henry et al. 2015; Wang et al. 2016; Zhang et al. 2016). Kawasaki et al. (2008) showed that TIMP-1, an endogenous inhibitor of MMP-9, is a powerful agent for suppressing neuropathic pain. We observed that the TIMP-1 level in DRG is significantly upregulated by LPS-RSU compared with that in INTACT and CCI-exposed animals. Moreover, the observed upregulation of TIMP-1 compared with its expression in the INTACT group was also significant in the spinal cord. This may suggest that LPS-RSU treatment stimulates antinociceptive TIMP-1 activation to oppose the CCI-induced upregulation of pronociceptive MMP-9.

## Conclusions

Pharmacological blockade of TLR4 diminished neuropathic pain behaviours. Moreover, we are the first to report that LPS-RSU, a highly specific TLR4 antagonist, modulated the nociceptive factors in DRG. LPS-RSU restored the balance between algesic IL-18 and analgesic IL-18BP, additionally increasing the IL-6 level, which in neuropathy is known to have analgesic properties. The change in the ratio between pro- (MMP-9) and antinociceptive (TIMP-1) factors, in favour of the latter may be one of the mechanisms of LPS-RSU-induced analgesia in neuropathy. Because most of the changes were detected in DRG, we hypothesize that LPS-RSU influences TLR4 expressed on IBA-1-positive cells, mainly macrophages; however, this needs to be clarified in the future. In our opinion, a better understanding of the role of TLR4 signalling in injury-induced pain will facilitate the development of neuropathy treatment.
